# Development and Validation of AAV-Mediated Liver, Liver-VAT, and Liver-Brain SORT and Therapeutic Regulation of FASN in Hepatic De Novo Lipogenesis

**DOI:** 10.3390/cells14050372

**Published:** 2025-03-04

**Authors:** Ratulananda Bhadury, Mohammad Athar, Pooja Mishra, Chayanika Gogoi, Shubham Sharma, Devram S. Ghorpade

**Affiliations:** Immuno-Inflammation Laboratory, National Institute of Immunology (BRIC-NII), Aruna Asaf Ali Marg, New Delhi 110067, India; ratul@nii.ac.in (R.B.); atharmehraj@nii.ac.in (M.A.); pooja2405mishra@gmail.com (P.M.); chayanika@nii.ac.in (C.G.); shubhambt2015@gmail.com (S.S.)

**Keywords:** adeno-associated virus (AAV), Selective ORgan Targeting (SORT), cysteine-rich with EGF-like domain 2 (CRELD2), fatty acid synthase (FASN), NASH

## Abstract

Hepatic lipogenesis combined with elevated endoplasmic reticulum (ER) stress is central to non-alcoholic steatohepatitis (NASH). However, the therapeutic targeting of key molecules is considerably less accomplished. Adeno-associated virus (AAV)-mediated gene therapies offer a new solution for various human ailments. Comprehensive bio-functional validation studies are essential to assess the impact of AAVs in the target organ for developing both preclinical and clinical gene therapy programs. Here, we have established a robust and efficient protocol for high-titer AAV production to enable detailed Selective ORgan Targeting (SORT) of AAV1, 5, 7, and 8 in vivo. Our results for in vivo SORT showed single organ (liver) targeting by AAV8, no organ targeting by AAV1, and dual organ transduction (liver-brain and liver-VAT) by AAV5 and AAV7. Using a human dataset and preclinical murine models of NASH, we identified an inverse correlation between ER stress-triggered CRELD2 and the de novo lipogenesis driver FASN. Furthermore, liver-specific silencing of CRELD2 via AAV8-shCreld2 strongly supports the contribution of CRELD2 to de novo lipogenesis through FASN regulation. Thus, our study demonstrates a robust method for producing clinically translatable AAVs that could be readily adapted for liver and/or liver-VAT or liver-brain targeted gene therapy.

## 1. Introduction

The hallmark of liver steatohepatitis is ER stress-mediated inflammatory response along with elevated hepatic lipogenesis [1,2]. Although an array of independent molecular regulators of ER stress inflammatory pathways and lipogenesis are identified, there exists a significant gap in our understanding of molecular regulators that link liver steatohepatitis to relevant ER stress and lipogenesis [3]. Recently, cysteine-rich with EGF-like domain 2 (CRELD2) has been uncovered as a novel ER stress-induced chaperon that holds the promise to regulate non-alcoholic steatohepatitis (NASH) [4]. However, the therapeutic potential of CRELD2 has been merely proposed but not yet validated. CRELD2 is ubiquitously expressed in multiple tissues, suggesting the need for an organ-specific delivery regime to maximize gene therapy’s potential [5]. In this regard, viral and non-viral vectors have been utilized to achieve organ-specific therapeutic gene targeting [6]. While Selective ORgan Targeting (SORT) has been explored to enhance non-viral lipid nanoparticle (LNP) specificity, it does not guarantee exclusive organ targeting [7,8]. As shown by Vaidya et al., LNPs achieve SORT in organs like the spleen and kidneys, but these nanoparticles still predominantly accumulate in the liver and other tissues [9]. Thus, the clinical use of LNPs for organ-specific drugging is limited. In contrast, adeno-associated viruses (AAVs) naturally possess tissue-specific targeting capabilities through their capsid proteins, with over 13 distinct variants exhibiting unique tissue tropism [10]. This inherent versatility makes AAVs ideal candidates for targeted gene therapies. Additionally, AAVs have a strong biosafety profile, exhibit stable and sustained gene expression, and elicit low immunogenicity—critical features needed for clinical applications [11]. As a result, numerous AAV-based therapies are already on the market, while over 200 therapies are currently in clinical trials across various therapeutic areas [12]. For example, preclinical studies have demonstrated the efficacy of AAV-delivered cargo in correcting genetic defects in animal models of diseases like muscular dystrophy, hypercholesterolemia, congenital amaurosis, hemophilia, and urea cycle disorders [13,14,15,16,17]. Researchers highlight that the biodistribution patterns of AAVs following in vivo administration play a critical role in defining their efficacy profiles in both preclinical and clinical settings, serving as a pivotal reference for predicting optimal human dosage [18,19]. However, emerging studies on cell receptor and AAV capsid interactions highlight that biodistribution data alone cannot pinpoint AAV uptake at the cellular level [20]. Such generalized tropism validation merely based on biodistribution may be inappropriate for evaluating AAVs transduction efficiency and alteration of AAV-based gene targeting. This limits the generation of reliable data for preclinical studies and impedes the advancement of AAVs as a therapeutic tool. Accordingly, a proper bio-functional validation necessitates an easy-to-reproduce and efficient AAV production protocol.

In the current study, we took two-pronged approaches to achieve organ-specific precision therapeutics. (1) We developed and optimized an efficient, scalable, and reproducible AAV production to validate in vivo preclinical bio-functional validation of AAV’s SORT. (2) We used our in-house-developed AAV8-shCreld2 tool to illustrate the regulatory role of liver CRELD2 in hepatic de novo lipogenesis. From an AAV production standpoint, we developed a simple and robust protocol for generating high-titer AAVs encapsulating self-complementary RNAi (sc-RNAi) vector targeting a gene of interest that can be easily adapted by any conventional laboratory worldwide. The key features of our protocol include the forward mode of triple transfection in HEK293T cells, combinatorial use of HDAC inhibitor and tryptone to enhance replication of the viral genome and accelerate production of viral proteins, collection of both secretory and cell-trapped AAVs, quick PEG-based precipitation of viral particles and rapid purification of high-titer AAVs without loss of bio-infectivity. The additional goal that we achieved in this study is AAV-sc-RNAi-mediated organ-specific gene silencing. To validate AAV’s SORT, we selected four different AAV capsid serotypes, namely, AAV1, AAV5, AAV7, and AAV8, and designed an AAV-sc-RNAi tool that encodes shRNA against CRELD2.

Our results demonstrate that AAV8 preferentially targets the liver, AAV7 transduces both liver and visceral adipose tissue (VAT), AAV5 shows dual tropism for the liver and brain, and AAV1 exhibits no tissue-specific targeting of CRELD2. Overall, our study has devised an improved, easy-to-recapitulate high-titer AAV production protocol, experimentally validated AAV’s capsid-dependent SORT. Moreover, liver-directed AAV8-shCreld2 therapy successfully elevated hepatic FASN expression, suggesting the key role of liver CRELD2 in linking hepatic ER stress and de no lipogenesis. This discovery enables researchers to test liver-, liver-VAT-, and liver-brain-directed AAV-mediated SORT gene therapy and raises the possibility of shortening the clinical path of translational discoveries.

## 2. Materials and Methods

### 2.1. Animal Experiments

All animals were housed in a specific pathogen-free facility under a 12/12 h light/dark cycle at ambient temperature (22 ± 2 °C) in 30–50% humidity with free access to a standard chow diet (Altromin, 1328, Lage, Germany) and water. All animals were acclimatized for 1 week in the animal facility before the commencement of experiments. For bio-functional validation of the AAV’s SORT, 6-weeks-old C57BL/6J male mice were injected with AAV-shCreld2 (1 × 10^15^ genome copies per mouse) or AAV-shScr (shRNA scramble as control) via tail vein. After 4 weeks of injection, mice were sacrificed, blood was collected through a cardiac puncture, and liver, VAT, colon, spleen, muscle, heart, lung, thymus, pancreas, brain, and kidney were isolated, snap frozen in liquid nitrogen and stored in −80 °C deep freezer for further analysis. For the relevant NASH murine model, four-week-old male C57BL6/J mice were fed with a fructose, palmitate, and cholesterol (FPC) diet (VRK nutritional solutions, Pune, India) (Supplementary Table S1) for 40 weeks [21]. The natural fasting-feeding experiment was conducted by fasting six-week-old C57BL/6J male mice overnight, followed by feeding for one hour with a standard chow diet. Mice were randomly distributed into experimental cohorts based on body weight and age. We did not predetermine the sample size; instead, we used group sizes that are typical for this type of study in the literature. The investigators were not blinded to the group allocations during the experiments and outcome assessments. Mice were excluded from the study if they exhibited skin lesions from fighting, growth retardation resulting in weight loss of 10% of their initial body weight, or showed signs of illness requiring euthanasia. Based on these criteria, typically, one and/or two mice were removed before the experimental set-up. All the animal studies were performed following the guidelines established by the Institute Animal Ethics Committee (IAEC) with approval number IAEC#590/22 on the use and care of animals at the National Institute of Immunology (BRIC-NII).

### 2.2. Quantitative Reverse Transcription–PCR (qRT-PCR) Analysis

Total RNA was extracted from tissue samples using TRIzol (G biosciences, 786-652, St. Louis, MO, USA). The concentration, purity, and quality of the isolated RNA were measured using Nanophotometer N120 (Implen, Munich, Germany). A total of two μg of RNA was reverse transcribed using Prime script first strand cDNA synthesis kit (Takara, 6110A, Kusatsu, Japan) using oligo dT as a primer. Real-time quantitative (q) PCR was performed with CFX opus 384 and CFX opus 96 (Bio-Rad, Hercules, CA, USA) using TB Green Premix Ex taq (Takara, RR420A, Kusatsu, Japan) and gene-specific primers. The relative expression levels were normalized to the expression levels of *36b4*. The sequences of gene-specific primers used for the qRT-PCR assays are enlisted in Table S2 (Supplementary Materials).

### 2.3. Immunoblotting

Protein lysates were prepared from organs using RIPA lysis buffer (G Biosciences, 786-490, St. Louis, MO, USA) supplemented with protease phosphatase inhibitor. Lysates were cleared by centrifugation using centrifuge 5424R (Eppendorf, Hamburg, Germany) at 15,000 rpm for 15 min at 4 °C. The protein content of lysates was measured using the DC Protein Assay kit (Bio-Rad, 5000112, Hercules, CA, USA) with a Clariostar plate reader (BMG Labtech, Ortenberg, Germany). An equal amount of protein was resolved along with a pre-stained protein ladder (Bio-Rad, 1610374, Hercules, CA, USA) on 10–15% SDS-PAGE gels and then transferred to PVDF membranes (Merck, IPVH00010, Berlin, Germany). The membranes were blocked at room temperature for one hour using 5% non-fat milk (Merck, 70166, Buchs, Switzerland) followed by incubation with primary antibodies overnight at 4 °C. Blots were then washed thoroughly with TBST and probed with HRP-conjugated secondary antibodies for one hour at room temperature. Protein bands were detected using the Clarity Western ECL substrate (Bio-Rad, 170-5061, Hercules, CA, USA) and imaged with the Image Quant 500 system (Amersham, Tokyo, Japan). To ensure equal protein loading, the same blot was stripped with stripping buffer (1.5% glycine (SRL, 69422, Mumbai, India) + 0.1% SDS (Sigma Aldrich, L3771, Tokyo, Japan), 0.1% Tween20 (Sigma Aldrich, P2287, St. Quentin Fallavier, France), pH 2.2) and then incubated with an HRP-conjugated anti-mouse GAPDH or β-ACTIN or HSP90 antibody. Relative intensities of protein bands were quantified using ImageJ (Version 2.14.0/1.64f, Bethesda, MD, USA) analysis software and normalized with the housekeeping proteins GAPDH, β-ACTIN, or HSP90. The antibodies used for the western blot analysis are enlisted in Table S3 (Supplementary Materials).

### 2.4. Annealing of shRNA Oligos

Single-stranded shRNA targeting *Creld2* and its complementary strand, corresponding to each clone listed in Supplementary Materials (Table S4), were dissolved in 1X T4 DNA ligase buffer (NEB, B0202S, Ipswich, MA, USA) to a concentration of one µg/µL. Equal amounts of each oligonucleotide were mixed, and the tubes were incubated in a Proflex PCR system (Applied Biosystems, Jurong, Singapore) set to 95 °C for fivemin. The temperature was then gradually decreased by 1 °C per minute until reaching 4 °C. Annealed oligos were stored at 4 °C until ligation with a digested vector.

### 2.5. Restriction Digestion of Vector and Ligation of shRNA Oligos to Make Recombinant Vector

The plasmid scAAV-H1-RSV-eGFP (933FF) was digested by incubatingone µg of vector DNA with one µL of BbsI enzyme (NEB, R0539L, Ipswich, MA, USA) at 37 °C for two h, followed by heat inactivation at 70 °C for 20 min. The digested DNA was then resolved on a 1% agarose gel, and the desired fragment was gel extracted using the manufacturer’s protocol (Qiagen, 28706, Hilden, Germany). Subsequently, 30 ng of the digested vector was ligated with 5 µL of the annealed oligo (above) in the presence of 10X T4 DNA ligase buffer (NEB, B0202S, Ipswich, MA, USA) and T4 DNA ligase (NEB, M0202S, Ipswich, MA, USA). The ligation reaction was carried out overnight at 16 °C, and the ligation mix was stored at 4 °C.

### 2.6. Preparation of Competent Cells

The Stbl3 strain of *E. coli* was inoculated in 10 mL of LB broth (HiMedia, G1245, Thane, India) and incubated overnight at 37 °C with shaking at 150 rpm. The following day, 500 µL of the overnight culture was added to 40 mL of LB medium and grown at 37 °C with shaking at 150 rpm, with measurement of optical density (O.D.) at 600 nm at regular intervals. Once the O.D. reached 0.35, bacterial growth was halted by placing the culture on ice for a short time. The cells were subsequently centrifuged at 8000 rpm for one min at 4 °C to collect the pellet. The bacterial pellet was then resuspended in 20 mL of transformation buffer (Table S5, Supplementary Materials) and kept on ice for 30 min [22]. *E. coli* cells were centrifuged after 30 min, and the pellet was dissolved in 3.6 mL of transformation buffer, followed by the addition of 400 µL of DMSO (Sigma Aldrich, D5879). *E. coli* competent cells were aliquoted in 100 µL volumes into pre-cooled microcentrifuge tubes, snap frozen in liquid nitrogen, and stored at −80 °C for further use.

### 2.7. Transformation of Recombinant Plasmids into Competent Cells

The competent cells were first thawed on ice. A total of five µL of KCM buffer (5X) (Table S6, Supplementary Materials) was added to the ligation mixture (above) [23]. The ligation mix with KCM buffer was mixed with competent *E. coli* cells and gently mixed by tapping the tube with a finger, avoiding pipetting up and down. The cells were then incubated on ice for 30 min. Following incubation, they were heat-shocked at 42 °C for 60 s and immediately placed back on ice for two–four min. A total of 400 µL of LB broth was added, and the cells were incubated at 37 °C for one hour to recover. After incubation, the cells were centrifuged at 5000 rpm for 5 min. Transformed *E. coli* cells were resuspended in the 200 µL of fresh LB broth. The resuspended cells were then plated using the spread plate method on Carbenicillin (HiMedia, PCT1102, Thane, India) containing LB Agar (HiMedia, M1151, Thane, India) and incubated overnight at 37 °C. A single colony of respective clones was picked and stored as glycerol stocks for future use.

### 2.8. Plasmid Isolation for Virus Production

Bacterial stocks containing different plasmids (AAV-shCreld2, AAV-Rep (2)/Cap (1, 5, 7, and 8), and Ad-Helper) were individually inoculated in terrific broth (Dr. Arie Geerlof, EMBL Hamburg, Germany) (Table S7, Supplementary Materials) and incubated overnight at 37 °C with shaking at 150 rpm. The following day, the bacterial cultures were centrifuged at 4 °C to pellet the cells and washed with PBS to remove any residual LB broth. Plasmid DNA was then isolated using the SurePrep Plasmid Midi Kit (Genetix, NP-15143, New Delhi, India), following the manufacturer’s protocol. The isolated plasmids were dissolved in 500 µL of Milli-Q water and stored at −80 °C for future use. The quality and concentration of the plasmids were assessed using a Nanophotometer N120 (Implen, Munich, Germany).

### 2.9. Cell Culture and Transfection

N2A cells (ATCC, CCL-131, Manassas, VA, USA) were used to validate the knockdown efficiency of different ShRNA clones targeting *Creld2* expression. Cells were maintained in DMEM high glucose (Sigma Aldrich, D6648, St. Louis, MO, USA), supplemented with 10% FBS (GIBCO, 10270-106, Sao Paulo, Brazil), 100 U/mL of penicillin, and 100 µg/mL of streptomycin (GIBCO, 15240-015, Carlsbad, CA, USA) at 37 °C with 5% CO_2_ in a humidified incubator. Transfection was carried out using Lipofectamine 3000 (Invitrogen, L3000-015, Vilnius, Lithuania) following the manufacturer’s protocol. After six h, the transfection media was changed to DMEM complete media (DMEM high glucose supplemented with 10% FBS, 100 U/mL of penicillin, and 100 µg/mL of streptomycin) and kept for 48 h. After 48 h post-transfection, cells were harvested. Total RNA and protein were isolated for qRT-PCR and western blot analysis, respectively.

### 2.10. Virus Preparation by Transfection

HEK293T cells (ATCC, CRL-3216, Manassas, VA, USA) were used to generate AAVs. Cells were cultured in DMEM high glucose, supplemented with 10% FBS, 100 U/mL of penicillin, and 100 µg/mL of streptomycin at 37 °C with 5% CO_2_ in a humidified incubator. On the day of transfection, a transfection mixture was prepared in a 1:3 ratio of DNA to PEI MAX (MW 40,000) (Polysciences, 24765, Brotherly Love, PA, USA). shCreld2 plasmid (6.6 µg per flask), pAAV2RepAAV8cap or pAAV2RepAAV7cap or pAAV2RepAAV5cap or pAAV2RepAAV1cap (10.2 µg per flask), and pAd-Helper (13.2 µg per flask) were mixed in 2.5 mL of PBS (per flask) in one tube, while 9 µL of PEI per flask (10 mg/mL in 0.2 N HCl (Thermo fisher scientific, Q29505, Mumbai, India) stock) was mixed in 2.5 mL of PBS (per flask) in a second tube. Both tubes were mixed together and vortexed thoroughly. The transfection mixture was then incubated at room temperature for 20 min. For reverse transfection, HEK293T cells were trypsinized, pelleted, and resuspended in serum-free low-glucose DMEM (Sigma Aldrich, D2902, St. Louis, MO, USA) media. A fresh T182.5 flask (VWR, 10062-864, St. Louis, MO, USA) was prepared with 11 mL of low-glucose, serum-free DMEM media. A total of five mL of the transfection mixture was added to the flask, followed by the addition of 20 × 10^6^ cells per flask. The contents of the flask were gently mixed, and the cells were incubated at 37 °C in a CO_2_ incubator for 24 h. After 24 h, the DNA: PEI-containing media was replaced with 18 mL of fresh low-glucose serum-free media. For forward transfection, HEK293T cells were grown overnight to achieve 80% confluency in complete media. The next day, cells were washed with serum-free media and cultured in 15 mL of serum-free low-glucose DMEM. A total of five mL of transfection mixture was added dropwise onto the cells with a gentle swirling of the flask followed by incubation for the next 24 h. After 24 h, the DNA: PEI-containing media was replaced with 18 mL of fresh low-glucose serum-free media and supplemented with 2 mL of 5% tryptone (HiMedia, CR014, Thane, India) and 40 µL of 2.5 M sodium butyrate (Sigma Aldrich, St. Louis, MO, 303410) per flask. GFP expression was monitored over the next 72 h post-transfection. The kinetics of virus particle production was assayed with immunofluorescence microscopy and using flow cytometry analysis for detection of the GFP signal of transfected HEK293T cells.

At 72 h post-transfection, the HEK293T cells were scraped using a cell scraper, and the entire supernatant was transferred to a 50 mL falcon tube. Cells were centrifuged at 3000× *g* for 10 min at room temperature (RT). The viral supernatant was then carefully collected into a new 50 mL falcon tube and incubated on ice. The cell pellet was resuspended in one mL of sterile PBS, mixed thoroughly with a one mL pipette tip, and vortexed for 20 s. This mixture was centrifuged at 3000× *g* for 10 min at RT. The cell-free supernatant was mixed with viral supernatant (collected above). A total of 5 mL of 40% PEG (Sigma Aldrich, P2139, St. Louis, MO, USA)—NaCl (HiMedia, GRM3954, Thane, India) solution (5×) was added to 20 mL of the combined supernatant. The mixture was inverted at least 20 times to ensure thorough mixing and then incubated on ice for two h. After two h, the falcon tubes were centrifuged at 4000× *g* for 30 min at 4 °C. After centrifugation, a white precipitate at the bottom of each tube was visible. The supernatant was carefully decanted without disturbing the pellet, and any remaining liquid was removed using one mL pipette. Next, 50 µL of ice-cold PBS (storage buffer) was added to the pellet (the amount depended on the pellet size). The pellet was mixed thoroughly using a pipette, and the resulting suspension was transferred to a sterile 1.5 mL microcentrifuge tube. The tubes were centrifuged at 10,000 rpm for five min at 4 °C. The top aqueous layer, containing the viral particles, was transferred to a new sterile tube, and the pellet (containing cellular proteins) was discarded. This centrifugation step (10,000 rpm for five min at 4 °C) was repeated to further remove cellular debris, and the supernatant containing the viral particles was collected in a new sterile 1.5 mL tube and stored at −80 °C for further analysis.

### 2.11. AAV Viral Titer Quantification

A total of 20 µL of the viral supernatant was aliquoted and diluted to 200 µL by adding 180 µL of PBS. The sample was sonicated at 50 Amp for 15 s, and AAV-ITR-PCR was performed using 1:10, 1:20, 1:40, and 1:80 dilutions of the sample. The viral titer was calculated in viral genome copies (GC) per mL (Calculation was given in an extended file). Simultaneously, the purity of viral preparation was assayed by loading viral lysate on SDS-PAGE gel followed by Coomassie blue staining or immunoblotting for the detection of viral capsid proteins, VP1, VP2, and VP3.

### 2.12. TEM Imaging

A total of five µL of the sample was loaded onto a Formvar square mesh, Cu grid (Electron microscopy sciences, FF300-CU, Hatfield, PA, USA), and kept for complete drying. The grid was negatively stained by 1.5% Uranyl Acetate (SRL, 81405, Mumbai, India) for one min. Excess stain was blotted dry, and the grid was allowed to air dry. Image acquisition was performed using a Tecnai G2 F20 microscope (FEI company, Hillsboro, OR, USA).

### 2.13. Flow Cytometry

Cells were trypsinized and resuspended in complete media. Cells were washed with ice-cold FACS buffer (1% FBS in PBS solution). Cells were passed through a cell strainer (VWR, 10199-655) and kept on ice. Cells were immediately analyzed using a BD FACSVerse flow cytometer (BD Biosciences, Franklin Lakes, NJ, USA). Data were analyzed using FlowJo software (v 10.10.0, BD Biosciences, Franklin Lakes, NJ, USA).

### 2.14. Blood and Serum Analysis

The complete blood count (CBC) profile was analyzed using Yumizen H500 (Horiba medicals, New Delhi, India). The serum was isolated by allowing the blood to clot for 30 min at room temperature (RT), followed by centrifugation at 4000 rpm for 20 min at 4 °C. The freshly separated serum was then analyzed for liver function tests like alanine aminotransferase (ALT) and aspartate aminotransferase (AST) levels, kidney function tests like blood urea and blood creatinine, serum and liver lipid profile using a coralyzer mini clinical analyzer (Coral, Goa, India).

### 2.15. Microarray Data Acquisition

The Gene Expression Omnibus (GEO) database is a publicly accessible repository for high-throughput gene sequencing data. We selected datasets for this study based on two criteria: (A) liver tissue samples from individuals clinically diagnosed with NASH, and (B) normal liver or samples from healthy participants as negative controls. Two datasets were chosen from the GEO database for analysis: GSE24807 and GSE17470. GEO2R, an R-based analysis tool available on the GEO website, was used to identify differentially expressed genes (DEGs) between the groups. For our analysis, we specifically focused on the expression of *Creld2* and *Fasn* associated with metabolic syndromes and compared its expression levels between NASH and healthy participants. A cut-off of log fold change > 0.5 and an adjusted *p*-value < 0.05 was applied, with the Benjamini and Hochberg method used for *p*-value adjustment.

### 2.16. Statistical Analysis

All results are presented as mean ± SD, with statistical significance denoted as follows: ns (non-significant), * = *p* < 0.05, ** = *p* < 0.01, *** = *p* < 0.001, and **** = *p* < 0.0001. Statistical analyses began with assessing data distribution using the Shapiro–Wilk test. For data with a Gaussian distribution, comparisons between two groups were performed using a two-tailed unpaired Student’s *t*-test, whereas non-Gaussian-distributed data were analyzed with the two-tailed Mann–Whitney test. For comparisons among multiple groups with Gaussian distribution, ordinary one-way ANOVA followed by Dunnett’s multiple comparison test was applied. For non-Gaussian data, the Kruskal–Wallis test, followed by Dunn’s multiple comparison test, was used. Correlation analyses were performed using Karl Pearson’s test for Gaussian-distributed data and Spearman’s rank test for non-Gaussian data. All statistical analyses were conducted using GraphPad Prism software (Version 10.2.1, San Diego, CA, USA).

## 3. Results

### 3.1. Construction of Self-Complementary RNAi Vector Against Creld2

**Figure 1:**
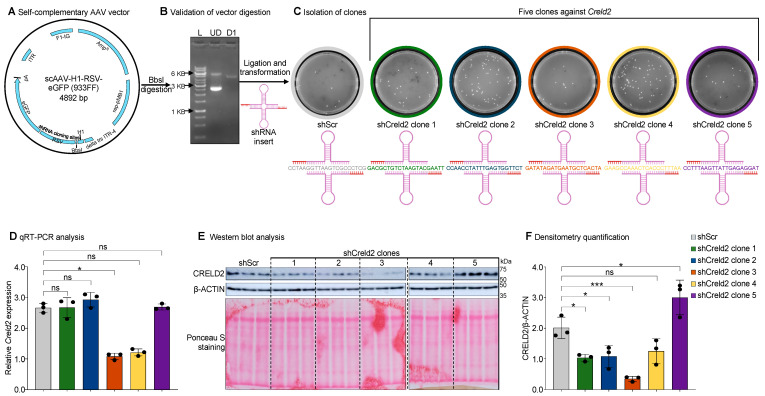
Construction and validation of sc-RNAi vector clones against *Creld2* for in vitro knockdown efficiency. (**A**) Vector map of scAAV-H1-RSV-eGFP plasmid showing shRNA cloning sites consisting of BbsI cut site. (**B**) Agarose image -post-scAAV-H1-RSV-eGFP plasmid BbsI-digestion. L, UD, and D1 denote ladder, un-digested, digested 1, respectively. (**C**) shScr clone and five clones of shCreld2 plasmid that target *Creld2* coding sequences. The sequences targeting the *Creld2* transcript are enlisted below every clone. (**D**–**F**) N2A cells were transfected with 5 AAV-shCreld2 clones along with AAV-shScr as control. The CRELD2 knockdown efficiency was evaluated using qRT-PCR (**D**), and Western blot analysis (**E**). The CRELD2 band intensity was quantified using Image J, and a graph of the relative expression of (β-ACTIN) was plotted (**F**). Data are shown as mean ± SD (n = 3) and were analyzed in (**D**) using the Kruskal–Wallis test followed by Benjamini and Hochberg multiple comparison test, and in (**F**) using an ordinary one-way ANOVA with Dunnett’s multiple comparison test (ns: non-significant, * *p* < 0.05, *** *p* < 0.001).

To validate the tissue tropism of AAVs, we constructed a self-complementary RNAi vector against *Creld2* as bait to observe the knockdown of druggable genes. We utilized a self-complementary (sc) AAV-H1-RSV-eGFP vector (Figure 1A) and ligated it with the corresponding shRNA sequence against *Creld2* (shCreld2) post-digestion with the enzyme BbsI (Figure 1B). For efficacious targeting, we designed five separate shRNA-oligonucleotides spanning different regions of the *Creld2* transcript (Figure 1C). After transfection of N2A cells with the five different clones of shCreld2, we observed that among five clones, clone 3 and clone 4 reduced *Creld2* expression by up to 60–70% (Figure 1D). To validate knockdown efficiency, we performed immunoblot analysis for the expression of CRELD2 and found shCreld2 clone 5 did not affect the silencing of CRELD2, shCreld2 clone 1, clone 2, and clone 4 suppressed CRELD2 expression by approximately 50%, while clone 3 could successfully knock down CRELD2 with an efficiency of over 90–95% (Figure 1E,F). We, therefore, selected shCreld2 clone 3 (hereafter referred to as shCreld2) for further crafting and optimization of the AAV-sc-RNAi tool.

### 3.2. Forward Transfection Exhibited Rapid GFP Expression than Reverse Transfection

**Figure 2:**
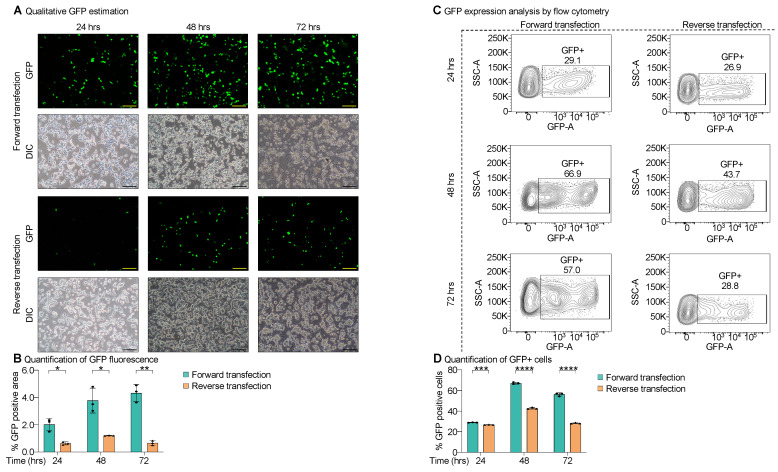
Forward transfection exhibited rapid GFP expression. (**A**,**B**) Representative images of GFP expression at 24, 48, and 72 h post forward and reverse transfection (**A**). The GFP intensities were quantified and plotted between forward and reverse transfection protocols (**B**). (**C**,**D**) Flow cytometry-GFP analysis of forward and reverse transfections along with quantification of GFP positive cells is plotted. Data are represented as mean ± SD (n = 3) and were analyzed using two-way mixed ANOVA with Tukey’s multiple comparison test (* *p* < 0.05, ** *p* < 0.01, *** *p* < 0.001, **** *p* < 0.0001). The scale bar is 100 μm.

We addressed the key parameters in the protocol to achieve high-titer AAV production. The pH of the production media is a critical factor in maintaining HEK293T producer cells [24]. The use of low-glucose DMEM media over high-glucose-containing DMEM has been shown to increase the titers of AAVs [24]. Another approach that may help in AAV production is facilitating the transcription of AAV capsid genes by host transcription machinery. Thus, 24 h post-transfection, the production media (low-glucose DMEM) was supplemented with 5 mM sodium butyrate (histone deacetylase inhibitor) and 0.5% tryptone to prolong the AAV capsid genes transcription and translation. One of the key aspects of high-titer virus production is the mode of transfection of producer cells. In the field, both forward and reverse transfection models are discussed without head-on comparisons. In this study, we compared and optimized the mode of transfection for high-titer AAV production. In the forward transfection approach, HEK293T cells are seeded 15 h before being triple-transfected with plasmids containing shCreld2 or shScr plasmid + AAV capsid 8/7/5/1 plasmid + Helper plasmid using polyethyleneimine (PEI). In contrast, reverse transfection entails the seeding of HEK293T cells and simultaneous addition of triple-transfection mixture (Figure S1). We assessed transfection efficiency by microscopically quantifying GFP expression at 24, 48, and 72 h post-transfection. We observed higher GFP expression in the forward transfection method compared to reverse transfection as early as 24 h post-transfection that continued to increase through 48 and 72 h post-transfection (Figure 2A,B). By 72 h, GFP expression was significantly higher in the forward transfection group compared to the reverse-transfected cells. To corroborate these findings, we conducted a flow cytometry analysis (Figure S3A,B). Almost 65% of the cell population tested GFP positive after 48 h of forward transfection compared to 40% GFP positive cells by reverse transfection (Figure 2C,D). Together, these data suggest that forward transfection is a superior method to achieve the highest transfection efficiency.

### 3.3. Purification of Virus by PEG-NaCl Method

**Figure 3:**
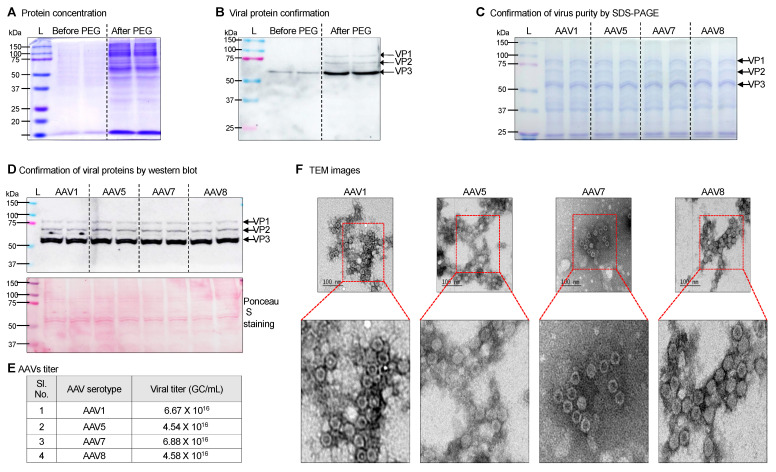
Harvesting and characterization of AAV-sc-RNAi tool. (**A**) Concentrated proteins after PEG precipitation were confirmed using Coomassie blue staining. (**B**) Concentration of virus particles after PEG precipitation was determined using western blotting. (**C**–**F**) High-titer AAV1-shCreld2, AAV5-shCreld2, AAV7-shCreld2, and AAV8-shCreld2 were lysed, and total proteins were resolved on SDS-PAGE gel, Coomassie blue stained (**C**), the AAV capsid VP1, VP2, and VP3 bands were detected using western blotting (**D**), viral titer was determined by q-PCR (**E**), and purity of AAV-sc-RNAi tool was visualized with TEM analysis (**F**). The scale bar is 100 nm.

Purification of AAVs poses a particular challenge as not all produced AAVs are released into the medium. Some were found to be trapped inside the AAV producer HEK293T cells. To address this, we centrifuged the HEK293T AAV producers along with the culture medium to collect the released viruses and concentrate the cell pellets. The cellular pellets were then resuspended in phosphate-buffered saline, resulting in the release of the majority of viral particles into the solution. These released viral particles were subsequently combined with the virus-enriched media supernatant, and AAVs were concentrated using PEG-NaCl precipitation. PEG precipitation followed by the centrifugation helped in the concentration of AAVs. However, the viral concentrate had HEK293T cellular contamination. The purification with density gradient ultracentrifugation in our hand led to a significant loss of AAV titer. However, a simple clarification step using centrifugation of resuspended AAV pellets into a smaller volume of PBS at 10,000 rpm for 5 min at 4 °C removed most of the cellular debris, and AAVs were concentrated in the supernatant (Figure S2). The concentrated viruses were confirmed with Coomassie staining and immunoblotting against viral VP1, VP2, and VP3 proteins (Figure 3A,B). Further, the purity of concentrated AAVs (serotypes 1, 5, 7, and 8) was determined by immunoblotting using anti-AAV capsid proteins VP1, VP2, and VP3 antibodies (Figure 3C,D). For the estimation of AAV genome copy number, we performed q-PCR using the primers specifically targeted to ITR regions of AAV. The titer was estimated using a standard curve obtained from the CT values vs. standard genome copies (Figure 3E). These data suggest that high-titer AAV production was achieved using the stated protocol irrespective of the AAV capsid serotypes. The details of viral titer calculation are summarized in the extended data file. Additionally, we imaged AAVs with transmission electron microscopy (TEM) and confirmed the viable icosahedral AAV production (Figure 3F). Collectively, we are able to concentrate AAVs via PEG-NaCl precipitation and obtain viable viruses with minimal cellular debris for bio-functional validation in mice.

### 3.4. Injection of High-Titer Virus in Mice Did Not Induce Any Toxicity or Affect Hepatic Physiology

To check for any toxicity of AAV at the high dose, we injected our in-house-produced high-titer AAVs (1 × 10^15^ GC per mouse) encapsulating shCreld2 vector via the mice tail vein to achieve whole-body distribution of AAV. In compliance with the ARRIVE guidelines, both shScr and shCreld2 virus-injected mice were closely monitored for toxicity at the experimental dose [25]. Mice were administered either AAV8-shScr, AAV8-shCreld2, or PBS as controls. No signs of toxicity, such as body weight change (Figure S4A), ataxia, hypoactivity, or piloerection, and no deaths were observed following intravenous (I.V.) injection up to a dose of 1 × 10^15^ GC per mouse, which was identified as the no-observed-adverse-effect level (NOAEL). Administration of AAVs in vivo did not result in systemic inflammatory response, as indicated by stable total white blood cell (WBC) counts (Figure S4B). Additionally, other hematological parameters, including total red blood cell (RBC) count, hemoglobin levels, and hematocrit (Figure S4C–E), were consistent between virus-injected mice and wild-type controls. Given the kidneys’ vulnerability to external agents, serum urea and creatinine levels were unchanged between PBS- and AAV-injected mouse cohorts (Figure S4F,G), suggesting no renal damage at the administered viral dose. Similarly, we observed no signs of hepatotoxicity, as evidenced by unchanged liver weight (Figure S4H), serum ALT and AST (Figure S4I,J) levels, which remained comparable across mice injected with AAV8-shScr, AAV8-shCreld2, and wild-type controls (PBS-injected). Furthermore, the cardinal feature of liver physiology to regulate lipid metabolism (hepatic and serum triglyceride and cholesterol levels) was unaltered between the PBS-injected and AAV-injected mice (Figure S5A,B,G,H). These findings were corroborated in AAV7- and AAV5-injected mice (Figure S5C–F,I,J). Taken together, these results conclude that a single dose of AAV injection at 1 × 10^15^ GC per mouse does not cause toxicity or alter hepatic or renal physiology.

### 3.5. Whole-Body Distribution of AAV8-shCreld2 Exhibited Liver-Specific Targeting

**Figure 4:**
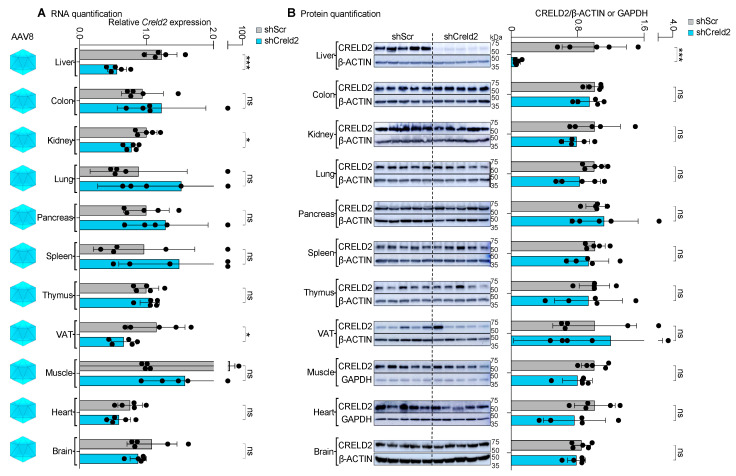
Whole-body distribution of AAV8-shCreld2 exhibited liver-specific targeting. (**A**) RNA quantification, (**B**) Protein quantification of CRELD2 of liver, colon, kidney, lung, pancreas, spleen, thymus, VAT, muscles, heart, and brain post AAV8-shScr and AAV8-shCreld2 injection. Band intensities were quantified using Image J and plotted relative to GAPDH or β-ACTIN levels. *36b4* was used as a housekeeping gene for all qRT-PCR analyses. Data are represented as mean ± SD (n = 5) and were analyzed using a two-tailed unpaired *t* test or Mann–Whitney test as appropriate (ns: non-significant, * *p* < 0.05, *** *p* < 0.001).

After confirming the absence of toxicity at the tested AAV dose, our primary goal was to validate the bio-functional capacity of the AAV cargo for achieving SORT in preclinical gene perturbation studies. To this end, we injected high-titer AAVs (1 × 10^15^ GC per mouse) containing the shCreld2 vector via the tail vein into mice to ensure whole-body distribution of AAV serotype 8. We then conducted gene expression analysis (both at RNA and protein levels) to assess the efficacy of the treatment. Given that previous studies have used the biodistribution profile of AAVs to determine their SORT profile, we further validated the functional capacity of the interference RNA (shRNA) technique to confirm the SORT outcome of AAVs. Among the various AAV capsids, AAV8 is the most widely studied, and in its natural form, it has been shown to be preferentially home to the liver. As discussed earlier, our optimized protocol is different yet simple than the commercially available cumbersome protocols, making it crucial to establish our own AAV titer capable of selectively knocking down genes in the liver using the AAV8 capsid. We optimized the dose kinetics of AAV8-mediated liver silencing and found that silencing of liver genes (*Creld2* and *Dpp4*) by AAV8 works best at a dose of 1 × 10^15^ GC per mouse (Figure S6A,B). Next, we assessed the tissue tropism of AAV serotype 8. We found that the liver CRELD2 levels were markedly reduced (93%) by shCreld2 when transduced by AAV serotype 8. The CRELD2 expression was unaltered in the other 10 tissues (AAV8-shScr vs. AAV8-shCreld2), suggesting AAV serotype 8 exhibits single organ tissue tropism (Figure 4A,B). To further validate the targeting of liver genes by AAV8-shRNAs, we constructed two additional RNAi vectors against *Dpp4* (AAV8-shDpp4) and *Hmgb2* (AAV8-shHmgb2). Consistent with the above data, we observed liver-specific knockdown of DPP4 and HMGB2 in mice cohorts injected with AAV8-shDpp4 and AAV8-shHmgb2, respectively (Figure S7A,B). Overall, our data affirm that AAV8 achieves liver-specific tropism.

### 3.6. Systemic Injection of AAV1-shCreld2 Did Not Exhibit Knockdown in Any of the Tested Tissues

**Figure 5:**
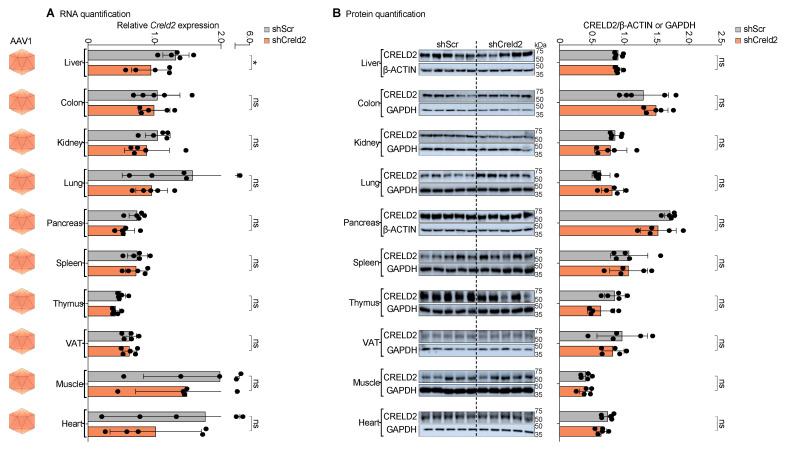
Systemic injection of AAV1-shCreld2 did not exhibit knockdown in any of the tested tissues. (**A**) RNA quantification, (**B**) Protein quantification of CRELD2 of liver, colon, kidney, lung, pancreas, spleen, thymus, VAT, muscles, and heart post AAV1-shScr and AAV1-shCreld2 injection. Band intensities were quantified using Image J and plotted relative to GAPDH or β-ACTIN levels. *36b4* was used as a housekeeping gene for all qRT-PCR analyses. Data are represented as mean ± SD (n = 5) and were analyzed using a two-tailed unpaired *t* test or Mann–Whitney test as appropriate (ns: non-significant, * *p* < 0.05).

After confirming that AAV8 specifically knocked down CRELD2 levels in the liver, we proceeded to validate the SORT profiles of other AAV serotypes. Following AAV8, AAV1 was the next serotype tested. We injected high-titer in-house-produced AAVs (1 × 10^15^ GC per mouse) containing the shCreld2 vector via the tail vein into mice to achieve whole-body distribution of AAV serotype 1, and subsequently performed *Creld2* gene expression analysis. The silencing of *Creld2* mRNA levels and CRELD2 protein expression in 10 different tissues, namely liver, colon, kidney, lung, pancreas, spleen, thymus, visceral adipose tissue (VAT), muscle and heart, is considered a SORT readout for the biological activity of AAV serotype infectivity. Systemic distribution of AAV1-shCreld2 did not alter the expression levels of CRELD2 in any of the 10 tissues (Figure 5A,B). These findings are critical as they highlight the limitation of validation of AAV SORT using labeled biodistribution techniques.

### 3.7. AAV5 and AAV7 Exhibited Dual Organ Targeting upon Systemic Distribution

**Figure 6:**
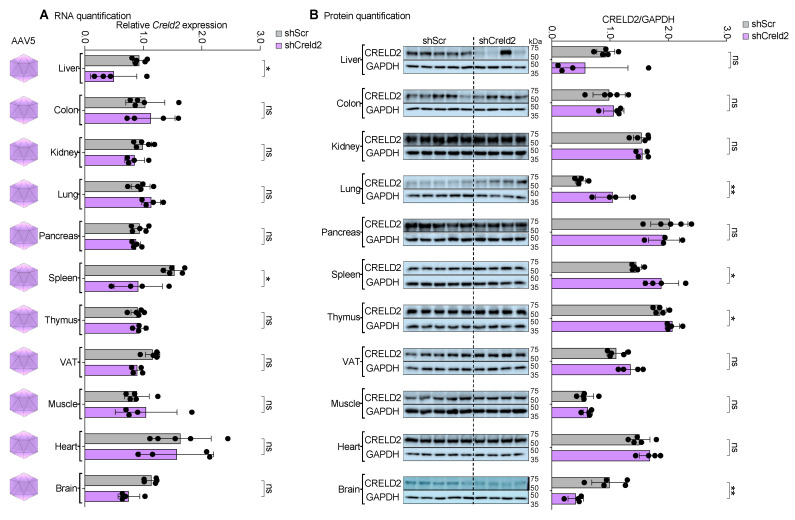
AAV5 revealed liver and brain targeting upon systemic distribution. (**A**) RNA quantification, (**B**) Protein quantification of CRELD2 of liver, colon, kidney, lung, pancreas, spleen, thymus, VAT, muscles, heart, and brain post AAV5-shScr and AAV5-shCreld2 injection. Band intensities were quantified using Image J and plotted relative to GAPDH levels. *36b4* was used as a housekeeping gene for all qRT-PCR analyses. Data are represented as mean ± SD (n = 4–5) and were analyzed using the two-tailed unpaired *t* test or Mann–Whitney test as appropriate (ns: non-significant, * *p* < 0.05, ** *p* < 0.01).

**Figure 7:**
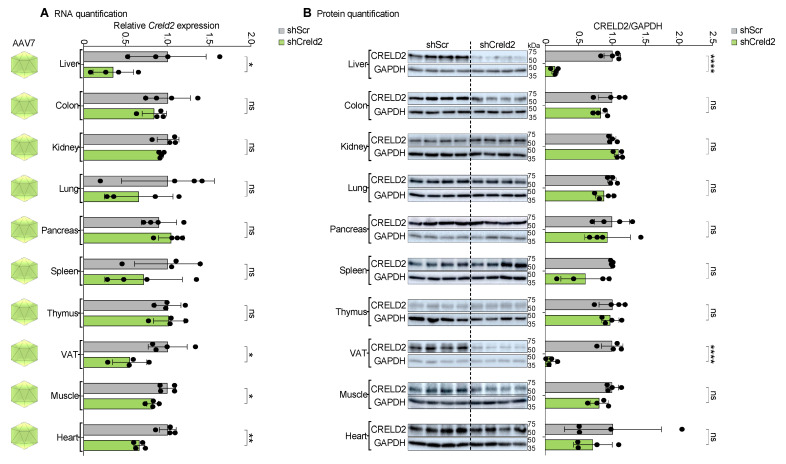
AAV7 displayed liver and VAT targeting upon systemic distribution. (**A**) RNA quantification, (**B**) Protein quantification of CRELD2 of liver, colon, kidney, lung, pancreas, spleen, thymus, VAT, muscles, and heart post AAV7-shScr and AAV7-shCreld2 injection. Band intensities were quantified using Image J and plotted relative to GAPDH levels. *36b4* was used as a housekeeping gene for all qRT-PCR analyses. Data are represented as mean ± SD (n = 4) and were analyzed using the two-tailed unpaired *t* test or Mann–Whitney test as appropriate (ns: non-significant, * *p* < 0.05, ** *p* < 0.01, **** *p* < 0.0001).

Our unexpected finding that AAV1 did not functionally target any of the tested tissues prompted us to further investigate the tissue tropism of other AAV serotypes. To this end, we separately injected AAV5-shCreld2 and AAV7-shCreld2 vectors into mice via the tail vein. Estimation of CRELD2 silencing efficiency in 11 different tissues underscored that AAV5-shCreld2 was able to silence liver and brain CRELD2 levels (38% and 59%, respectively), generating a possibility of studying the neuro-endocrine contribution of genes by altering their function in both liver and brain tissues at the same time (Figure 6A,B). The overall liver-specific silencing efficiency of AAV5 was less than 50% due to a biological variability observed in one mouse of the cohort. Interestingly, AAV7-shCreld2 particles could successfully silence both liver and VAT CRELD2 expression levels (86% and 92%, respectively). The simultaneous lowering of gene expression in liver and VAT by AAV serotype 7 establishes a unique tool for dual organ (liver and VAT) targeting (Figure 7A,B). Collectively, our data provide direct proof of AAV’s dual organ homing to the liver and brain (AAV5) and liver and VAT (AAV7) tissues.

### 3.8. Bio-Functional Evaluation of AAV8-shCreld2 in Models of Dyslipidemia

**Figure 8:**
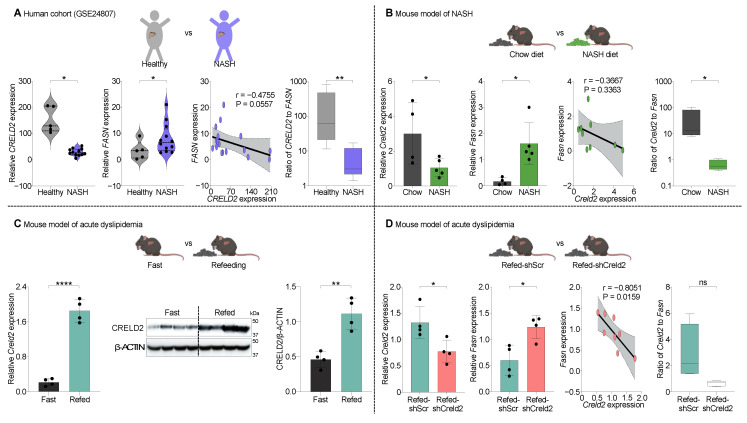
Bio-functional evaluation of AAV8-shCreld2 in models of dyslipidemia. (**A**) *CRELD2*, *FASN* gene expression along with their correlation analysis in the liver of healthy (n = 5) vs. NASH (n = 12) human patients. *p*-value < 0.05 with the Benjamini and Hochberg method. The ratio of *CRELD2* and *FASN* was plotted in log scale. (**B**) *Creld2*, *Fasn* gene expression along with their correlation analysis in the liver of mice fed with standard chow diet vs. NASH diet (n = 4–5). Ratio of *Creld2* and *Fasn* was plotted in log scale. (**C**) *Creld2* expression analysis both at transcript and protein levels with densiometric quantification in liver of mice that were overnight fasted vs. refed for 1 h (n = 4). (**D**) *Creld2*, *Fasn* gene expression along with their correlation analysis in liver of AAV8-shScr vs. AAV8-shCreld2 mice that were 1 h fed after overnight fasting (n = 4). Ratio of *Creld2* and *Fasn* was plotted. Data are represented as mean ± SD and were analyzed using the two-tailed unpaired *t* test or Mann–Whitney test, Pearson or Spearman correlation analysis as appropriate (ns: non-significant, * *p* < 0.05, ** *p* < 0.01, **** *p* < 0.0001).

CRELD2 is an ER stress inducible chaperone molecule that has been implicated in metabolic syndromes [26]. A study by Kern et al. has particularly discovered the importance of CRELD2 in liver steatosis [4]. However, their approach was to knock down the functions of CRELD2 throughout the body. This raises the question of the autocrine vs. endocrine functions of CRELD2 in regulating liver steatosis. We propose that using AAV8-shCreld2 mediated liver-specific silencing of CRELD2 would be a key to resolving this conundrum. Accordingly, we analyzed human datasets from the Gene Expression Omnibus (GEO) pertaining to non-alcoholic steatohepatitis (NASH). Our analysis revealed that *CRELD2* expression was significantly downregulated in NASH patients compared to healthy controls. Notably, *FASN*, a key enzyme driving de novo lipogenesis, exhibited a negative correlation with hepatic *CRELD2* expression in the liver of NASH patients. Quantitative analysis of the inverse correlation between *CRELD2 and FASN* revealed a significantly higher *CRELD2* to *FASN* ratio in healthy participants compared to those with NASH patients (Figure 8A and Figure S8). To corroborate these findings, we established a human-relevant NASH murine model by feeding mice with a NASH diet (Fructose Palmitate Cholesterol (FPC) diet). Consistent with human data, we observed an inverse correlation between *Creld2* and *Fasn* expression and a higher *Creld2* to *Fasn* ratio in the livers obtained from control mice compared to NASH mice livers (Figure 8B). Furthermore, we evaluated the expression of CRELD2 in the murine model of fasting followed by refeeding (acute de novo lipogenesis) and found that liver *Creld2* transcript and CRELD2 protein levels were markedly elevated in the refed mouse cohort (Figure 8C). To evaluate the potential of liver CRELD2 in de novo lipogenesis via *Fasn* regulation, we specifically knocked down CRELD2 in the liver using AAV8-shCreld2 and subjected mice to a temporal fasting-refeeding regime. The expression of liver *Fasn* in AAV8-shCreld2 injected mice was markedly increased, establishing the significant inverse correlation of liver *Creld2* to *Fasn* expression. Although a biological trend was observed with a higher *Creld2* to *Fasn* ratio in shScr refed mice compared to shCreld2 refed mice, this difference was not statistically significant (Figure 8D). We also analyzed other genes involved in the lipogenesis pathway and observed no changes in *Acc1*, *Srebp1*, *Fabp5*, and *Cpt1a* expression (Figure S9A). ShCreld2 mice exhibited nearly a twofold increase in liver triglyceride levels, although this difference was not statistically significant, while serum triglyceride levels remained comparable to those of shScr mice following refeeding (Figure S9B,C). Further investigation into liver fibrosis markers and inflammatory gene expression revealed no significant differences between wild-type (WT) and CRELD2-silenced mice (Figure S9D,E). The histopathological assessment indicated no additional immune cell infiltration in the liver, and lipid droplets were absent in these mice (Figure S9F). Despite these unchanged parameters in acutely fasted and refed mice, the elevated levels of *Fasn* observed in the livers of shCreld2 mice were associated with increased liver triglyceride levels. This suggests a potential link between CRELD2 and hypertriglyceridemia through the regulation of *Fasn*. The modulation of *Fasn* by liver CRELD2 may play a crucial role in the dyslipidemia associated with NAFLD-NASH phenotypes, warranting further investigation in future studies. These data are consistent with human and preclinical mouse models of NASH (above), thus providing strong support to our thought that using the AAV-sc-RNAi tool is the key to aiding the basic discoveries and suggests AAV-dependent SORT may be a stepping stone in the discovery and development of organ-specific therapeutics.

## 4. Discussion

Genes exhibit tissue-specific functions, and systemic therapeutic drugging may inadvertently disrupt these functions in non-target tissues, potentially leading to adverse off-target effects [27]. Thus, new technologies are needed to deliver therapeutic agents specifically to curb organ-specific genes that drive pathology. In this regard, AAVs have recently been discussed for delivering cargo to specific organs due to their capsid tissue tropism property [28]. For example, the FDA has approved the use of Hemgenix for the treatment of hemophilia, which is a drug composed of AAV serotype 5 (capsid-type-5) carrying gene factor IX to the liver [29]. Likewise, Zolgensma, a spinal muscular atrophy drug, is an AAV serotype 9 (capsid-type-9) that carries the survival motor neuron 1 (SMN1) gene to neurons via crossing the blood-brain barrier [30]. Despite the successful approval of such a few drugs, the efficacious delivery of AAV cargo to desired target cells and/or organs is yet to be validated. This hampers the development of precise medication for many diseases including rare ones. Therefore, a top priority is to carefully design a recombinant “AAV-based tool” and validate its SORT. To address efficient delivery of AAV-cargoes to target organs, we utilized AAVs with four different AAV serotype plasmid constructs (AAV serotype 8 (Capsid-type-8), AAV serotype 7 (Capsid-type-7), AAV serotype 5 (Capsid-type-5), and AAV serotype 1 (Capsid-type-1)). The current AAV production protocols are tedious, labor-intensive, and expensive, which hurdles the improvement of SORT gene editing applications [31,32,33,34]. Therefore, in the current study, we prioritize the development and optimization of a streamlined protocol for the high-titer production of AAVs, enabling effective in vivo SORT.

Efficient and high-titer AAV production is influenced by several key factors, including the quality of plasmid vectors, producer cells, transfection method, and the subsequent harvesting and purification of the virus [35,36,37]. In contrast to single-stranded AAV vectors, scAAVs circumvent the requirement for conversion to double-stranded DNA (dsDNA) by host cell machinery, thereby allowing for a more direct and effective delivery of the genetic material, facilitating improved performance in gene transfer applications [38]. We carefully designed short hair-loop oligonucleotides against *Creld2* transcripts as bait to evaluate the biological silencing of CRELD2 in multiple organs. Human embryonic kidney (HEK293) cells are commonly used for the production of therapeutic proteins and viral vectors for gene therapy due to their ease of culture, high transfection efficiency, and robust virus production [39]. In our study, we found that HEK293T cells are the robust producer of higher-titer AAVs. After careful optimization, we identified a polyethyleneimine (PEI): DNA ratio of 1:3 in a forward mode of transfection protocol yields maximized transfection efficiency. Cell health and culture conditions play a crucial role in high-titer AAV production [37]. The expression of viral proteins is closely linked to increased glycolysis and acid production, leading to a decrease in media pH, which can negatively impact HEK293T cell-mediated AAV production [24]. Previous studies have shown that the use of peptones can enhance protein expression, and the inhibition of histone deacetylases (HDAC) has been demonstrated to increase both antibody and AAV capsid production [40,41]. Thus, we supplemented AAV producer cells with 5 mM sodium butyrate (an HDAC inhibitor) and 0.5% tryptone. Collectively, these combined modifications led to the development of improvised high-titer AAV production. While our established protocol is designed to be highly adaptable for laboratories of various sizes, there are a few key highlights that require careful attention: (1) utilizing early passage HEK293T producer cells, (2) using fresh PEI, (3) ensuring proper reconstitution of concentrated viruses, and (4) performing a proper tail vein puncture to access systemic circulation.

For in vivo applications, a purified AAV is needed to achieve the biological gene editing effect. Previous studies have emphasized the importance of virus storage buffers and density gradient ultracentrifugation for the efficient harvesting and purification of AAVs [42,43]. In our study, we found that simply resuspending the PEG-concentrated virus in a small volume of PBS, followed by brief centrifugation, was sufficient for obtaining functional AAVs. Notably, these optimizations have retained the high titer of AAVs, circumventing the viral loss due to ultracentrifugation. AAVs exhibit natural adsorption to organs due to their icosahedral capsids, making them an excellent choice to validate in vivo SORT [44]. Multiple serotypes of AAVs are distinguished from the variability among the capsid organizations. There is an overall 45% similarity among AAVs capsid protein, and differences could contribute to tissue tropism [45]. Several studies have tried to evaluate the role of capsid variability in tissue-specific transduction. For example, serotypes 1, 2, and 5 infect different regions of the neuronal system with variable efficiencies. The role of serotypes 1, 2, 5, 7, and 8 is comparatively studied in a mouse brain, targeting of mouse lung by AAVs serotypes 2, 3, and 6, liver-specific silencing of genes using AAV serotype 8, muscle-specific gene delivery by AAV serotype 6, and AAV serotype 9 transduces cardiac muscles [46,47,48]. The body of research on AAV tissue tropism is complicated by considerable variability, with one of the major confounding factors being the route of delivery [45]. Systemic and localized injections exhibit distinct homing capacities, further complicating tissue tropism in vivo [49]. To enable more meaningful comparisons, it is essential to use the same delivery route—ideally a systemic one—across all serotypes. This approach aids in selecting the most suitable serotype for targeted gene delivery and provides a reliable basis for comparisons in animal models. One such study by Zincarelli et al. used the tail vein injection route to administer AAV and examined the transgene luciferase expression [45]. Additionally, many studies on AAV tropism focus on modifying the capsid protein to create recombinant AAV (r-AAV) vectors, with the goal of enhancing or broadening tissue homing capabilities [50,51]. However, the tissue tropism of wild-type AAV capsid proteins has not been fully validated. While a few studies have attempted to address these issues by investigating the spatial biodistribution of AAV serotypes, they often fail to provide functional validation of gene perturbation through the AAV payload [45,52]. Our findings highlight the limitations of studies that rely exclusively on fluorescent or radiolabeled probes to assess AAV biodistribution as a measure of tissue tropism. In our current study, by utilizing AAV-shCreld2 cargo, we biologically validate liver targeting by AAV8 viruses, simultaneous delivery of shCreld2 cargo to the liver and VAT via AAV7, and silencing of CRELD2 in the liver and brain by AAV5-shCreld2. Interestingly, while AAV1 is known to exhibit tropism for tissues such as the lungs, CNS, pancreas, and muscle, our study highlights that the mere presence of AAV1 in these tissues does not guarantee the desired biological effect of the delivered cargo [53,54,55,56]. Therefore, our findings emphasize that achieving the intended functionality of the delivered cargo is critical for effective SORT via AAVs, positioning our approach as a promising strategy for both therapeutic applications and genetic perturbation.

After validating the efficient AAV-mediated SORT in vivo, it is crucial to investigate the phenotype associated with the AAV payload. Since CRELD2 is an ER stress-associated gene that has been proposed as a biomarker for diseases linked to ER stress like NASH, diabetes, kidney disease, CVDs, and cancers, we extended our study to provide the proof of concept for bio-functionally validating the AAV-RNAi tool in a disease model of metabolic syndrome [22,57,58]. Evaluation of liver samples from patients with NASH and in FPC-fed mice revealed an inverse correlation between liver *Creld2* and lipogenesis gene *Fasn.* Selective targeting of liver CRELD2 via AAV8-shCreld2 in mice confirmed this relationship in an acute model of dyslipidemia. Our observation is consistent with a report that suggests CRELD2 knockout promotes the NASH phenotype in preclinical murine models [4]. This highlights the specific utility of AAV cargo in uncovering the paradigms that may be operative in human pathologies as well. While we have identified a heretofore unknown relationship of liver CRELD2 with *Fasn,* we acknowledge the scope for further detailed investigation into the role of hepatic CRELD2 in suppressing de novo lipogenesis. Our findings highlight the potential of bio-functional validation of the remaining AAV capsids to expand these tools for the development of organ-specific therapies aimed at addressing the complex pathophysiology underlying human diseases.

## 5. Conclusions

Although the systemic administration of medicines is widely used, it has side effects or off-target effects, and thus, precise organ drug delivery is preferred as one of the strategies for evolving biotherapeutics. However, the lack of platforms to deliver drug cargo in an organ-specific manner is one of the major hurdles in translating potential drugs to patients in need. In this study, we validated the proposed tissue tropism of 4 major AAV capsids in delivering RNAi cargoes. We developed and optimized the high-titer AAV production protocol that can be easily translated to achieve in vivo SORT. Exploiting tissue tropism property of AAVs, we first time validated AAV’s SORT to liver (AAV8), liver-VAT (AAV7), and liver-brain tissues (AAV5). Furthermore, we established liver CRELD2’s role in hepatic de novo lipogenesis using AAV8-shCreld2. This enabled us to unravel the previously masked role of liver CRELD2 in hepatic lipogenesis during the feeding phase with its clinical relevance to NASH. With our discovery, we could successfully link ER stress-responsive CRELD2 as an upstream regulator to the hepatic lipogenesis pathway. Accordingly, compromised functions of liver CRELD2 are evident in human NASH datasets and mouse models of NASH. We envisage that the carefully crafted and validated AAV-sc-RNAi tool in this study would have broader applications in the delivery of drug cargoes to desired organs with minimal adverse effects.

## Data Availability

The data that support the findings of this study are available within the article and Supplemental Materials or upon reasonable request from the corresponding author, D.S.G.

## Supplementary Materials

The following supporting information can be downloaded at: https://www.mdpi.com/article/10.3390/cells14050372/s1, Figure S1: Schematic diagram of forward and reverse transfection protocol in HEK293T cells for high-titer virus production; Figure S2: Schematic diagram of PEG-NaCl precipitation for high-titer virus purification; Figure S3: Representative flow cytometry gating strategy and quantification of relative cell populations; Figure S4: High-titer AAV injection does not exhibit any toxicity in mice; Figure S5: High-titer virus injections and silencing of liver CRELD2 did not change lipid profile of liver and serum at the basal level in chow diet fed mice; Figure S6: Dose kinetics of AAV8 mediated liver targeting; Figure S7: Validation of AAV8-sc-RNAi tool with shDpp4 and shHmgb2 plasmid; Figure S8: Negative correlation between liver *CRELD2* and *FASN* in human NASH patients; Figure S9: Comparison of AAV8 treatment in basic physiology of fast-refed mice; Table S1: Composition of FPC diet; Table S2: List of mouse primers used for qRT-PCR analysis; Table S3: List of antibodies used for western blot analysis; Table S4: List of all five Sh-Creld2 clone sequences; Table S5: Composition of transformation buffer; Table S6: Composition of KCM buffer (5×); Table S7: Composition of Terrific broth.
