# Supplementary material for: Development and Validation of AAV-Mediated Liver, Liver-VAT, and Liver-Brain SORT and Therapeutic Regulation of FASN in Hepatic De Novo Lipogenesis
Source: Cells. 2025 Mar 4;14(5):372. doi: 10.3390/cells14050372 (PMC11899426; doi:10.3390/cells14050372)
Supplement: Supplementary file 1 [file cells-14-00372-s001.zip › Supplementary materials- Figures S1-S9. Tables S1-S7.pdf]

Supplementary materials

Development and validation of AAV-mediated liver, liver-VAT and liver-brain therapeutic regulation of FASN in hepatic de novo lipogenesis

Ratulananda Bhadury, Mohammad Athar, Pooja Mishra, Chayanika Gogoi, Shubham Sharma and Devram S. Ghorpade *

Immuno-Inflammation Laboratory, National Institute of Immunology (BRIC-NII), Aruna Asaf Ali Marg,
New Delhi 110067, India; ratul@nii.ac.in (R.B.); atharmehraj@nii.ac.in (M.A.); pooja2405mishra@gmail.com (P.M.); chayanika@nii.ac.in (C.G.); shubhambt2015@gmail.com (S.S.)
* Correspondence: dev@nii.ac.in

Supplementary Figures

Figure S1

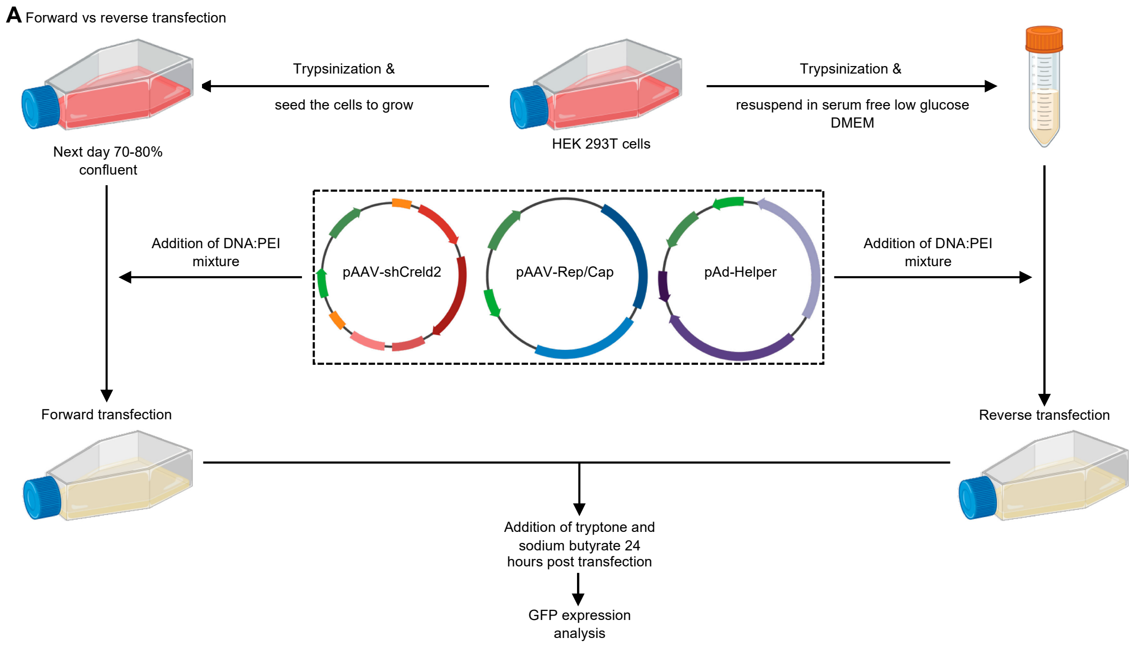

Figure S1. Schematic diagram of forward and reverse transfection protocol in HEK293T cells for high titer virus production. A) HEK293T cells were subjected to either forward transfection or reverse using a triple-plasmid-PEI transfection mixture in serum-free low glucose DMEM media, and transfected HEK293T cells were monitored for GFP expression over 72 hours.

Figure S2

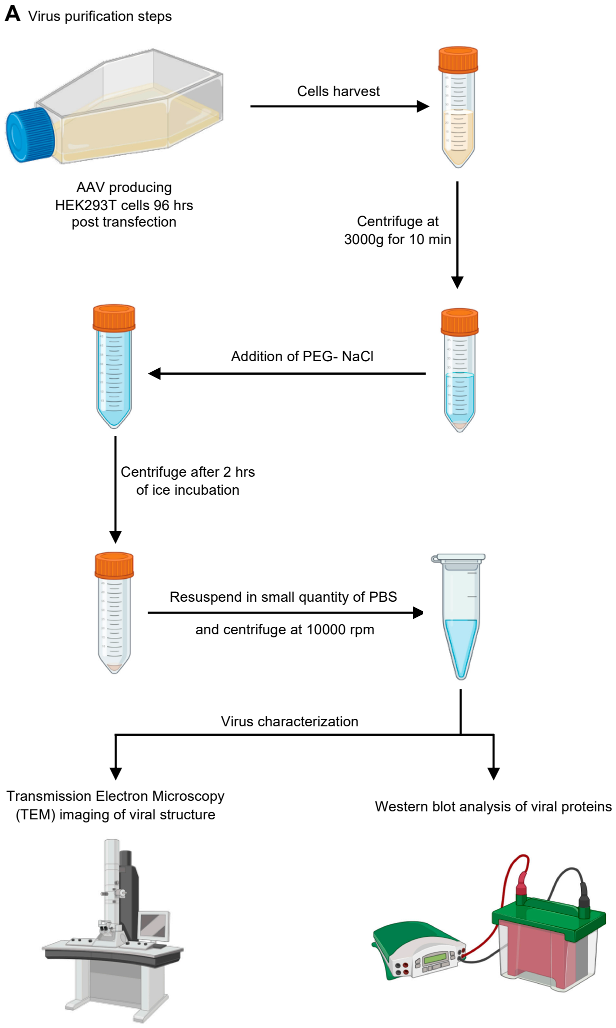

Figure S2. Schematic diagram of PEG-NaCl precipitation for high titer virus purification. A) HEK293T cells were harvested and virus was precipitated using PEG-NaCl. After 2 hrs ice incubation, a simple centrifugation step was performed to obtain high titer AAVs.

Figure S3

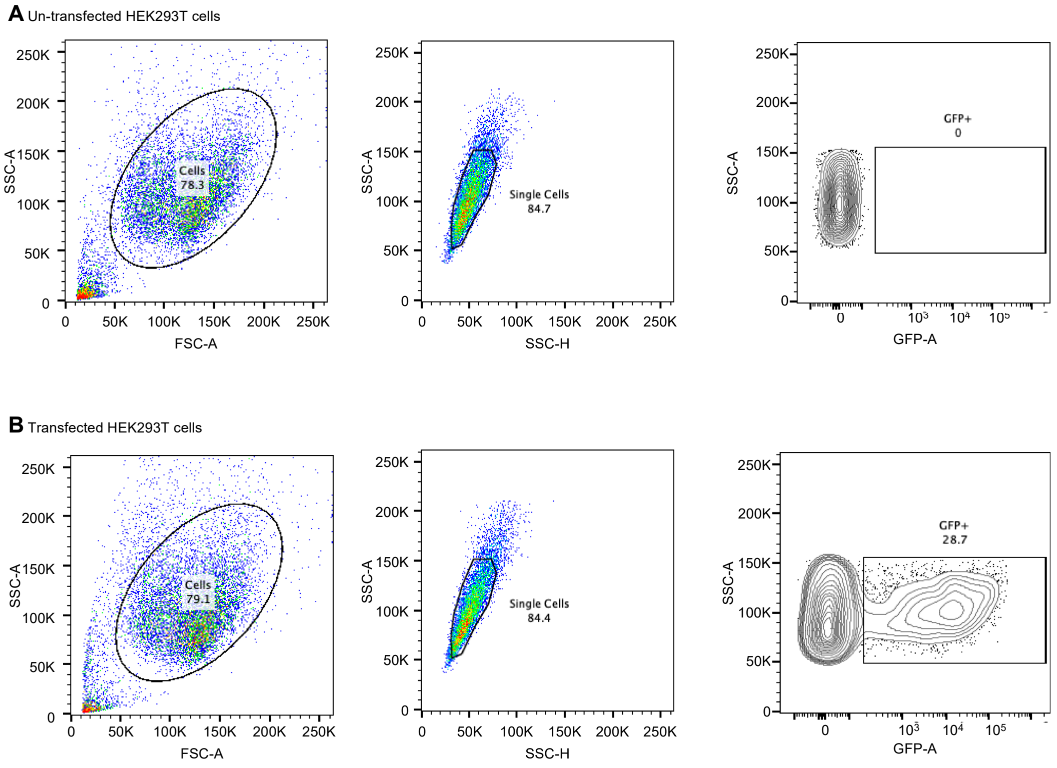

Figure S3. Representative flow cytometry gating strategy and quantification of relative cell populations for A) un-transfected HEK293T cells, B) transfected HEK293T cells.

Figure S4

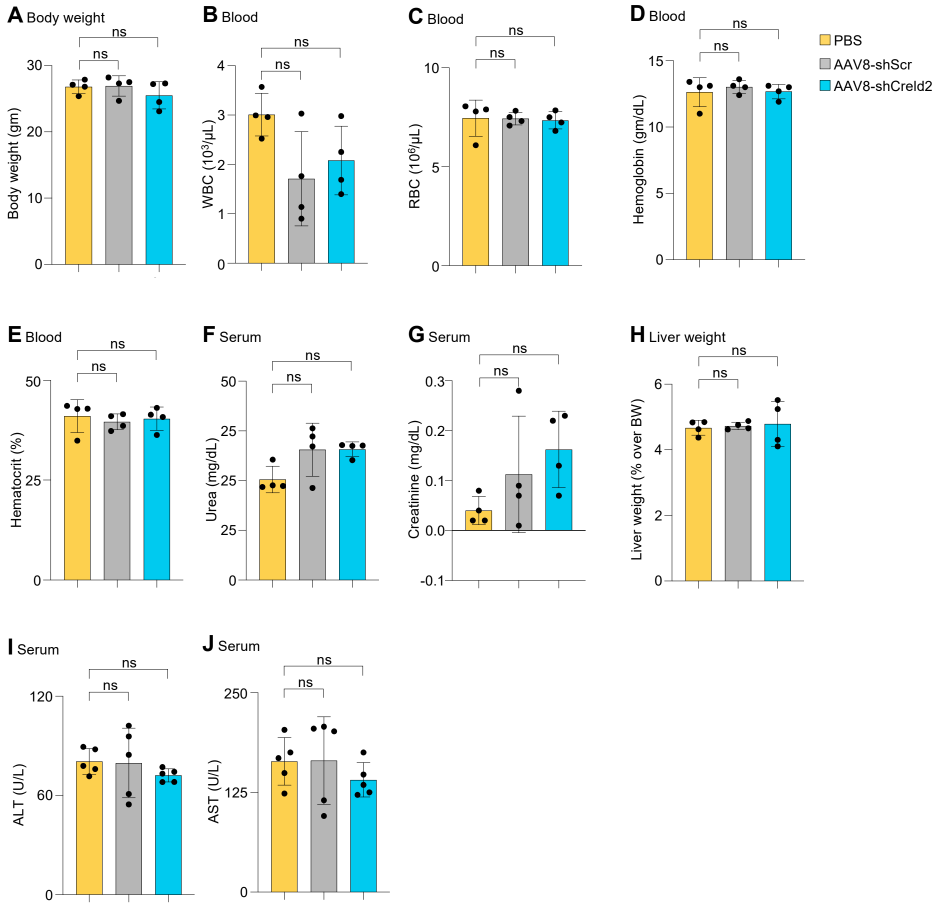

Figure S4. High titer AAV injection does not exhibit any toxicity in mice. Mice were injected intravenously with AAV8-shScr, or AAV8-shCreld2, or PBS as control. After 4 weeks, the following parameters were assessed to evaluate toxicity in mice: A) Body weight, B) total WBC count, C) total RBC count, D) hemoglobin level in blood, E) hematocrit level in blood, F) total urea level in serum, G) total creatinine level in serum, H) liver weight, I) serum ALT level, J) serum AST level. Data are shown as mean ± SD (n = 4-5) and were analyzed in (A, B, D, G, H, I) by ordinary one-way ANOVA with Dunnett's multiple comparison test, and in (C, E, F, J) by Kruskal-Wallis test followed by Dunn's multiple comparison test (ns: non-significant).

Figure S5

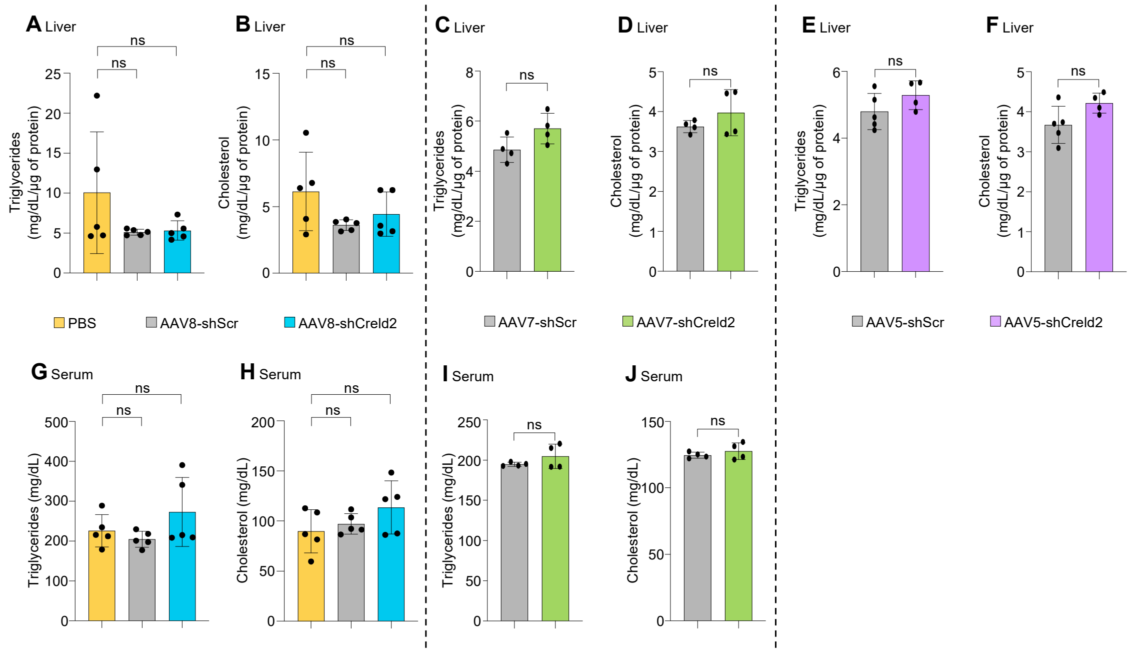

Figure S5. High titer virus injections and silencing of liver CRELD2 did not change lipid profile of liver and serum at the basal level in chow diet fed mice. A, B) Liver triglycerides and cholesterol levels in the mice injected with AAV serotype 8 compared to PBS injected cohort. C- F) Liver triglycerides and cholesterol levels in the mice injected with AAV7 and AAV5 particles. G, H) Serum triglycerides and cholesterol levels in the mice injected with AAV serotype 8 compared to PBS injected cohort. I, J) Serum triglycerides and cholesterol levels in the mice injected with AAV7 particles. Data are shown as mean ± SD (n = 4-5) and were analyzed by two-tailed unpaired t-test or Mann-Whitney test for two group and one-way ANOVA or Kruskal-Wallis test as appropriate (ns: non-significant).

Figure S6

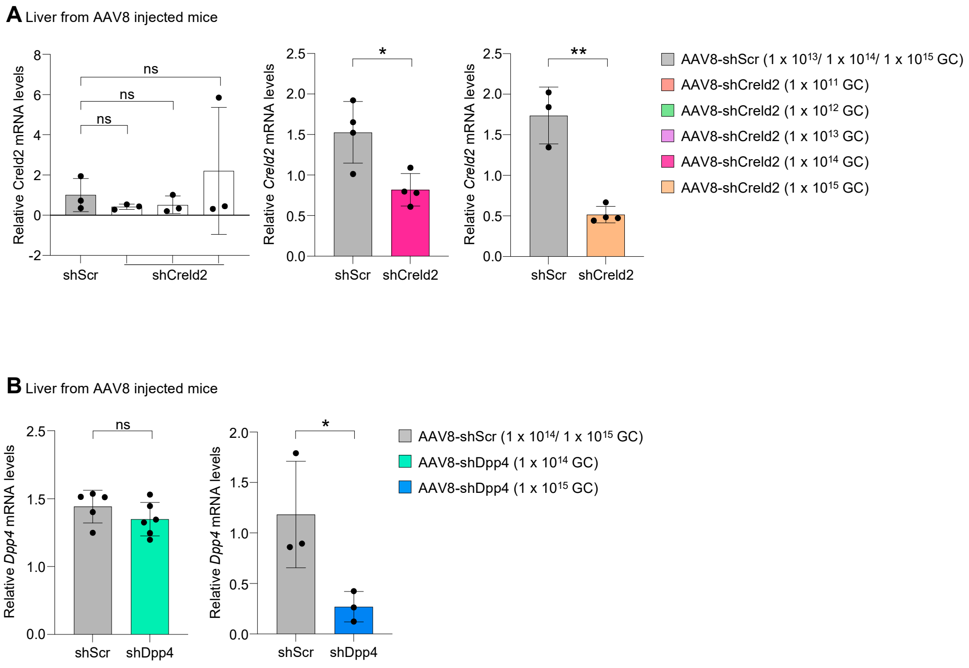

Figure S6. Dose kinetics of AAV8 mediated liver targeting. A) Relative *Creld2* expression levels in the liver of mice injected with AAV8-shScr, and/or AAV8-shCreld2 particles at a dose from 1 x 10^{11} to 1 x 10^{15} GC per mouse. B) Relative *Dpp4* expression levels in the liver of mice injected with AAV8-shScr, and/or AAV8-shDpp4 particles at a dose from 1 x 10^{14} to 1 x 10^{15} GC per mouse. *36b4* is used in all qRT-PCR experiments as housekeeping gene. Data are shown as mean ± SD (n = 3- 5) and were analyzed by Kruskal-Wallis test followed by Dunn's multiple comparison test for multiple groups, by Mann-Whiney test, and by unpaired t-test for two groups as appropriate (ns: non-significant, *p < 0.05, **p<0.01).

Figure S7

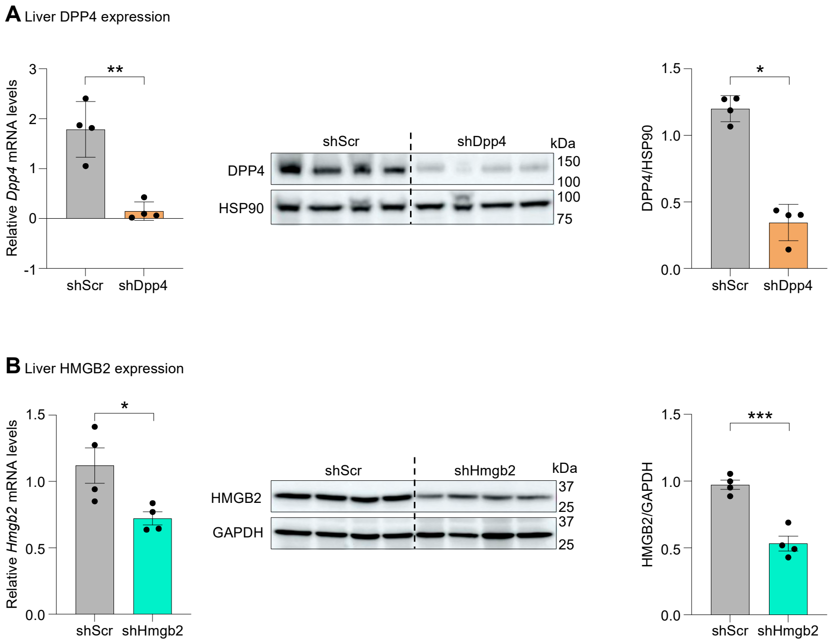

Figure S7. Validation of AAV8-sc-RNAi tool with shDpp4 and shHmgb2 plasmid. A) DPP4 expression analysis from liver of AAV8-shDpp4 vs AAV8-shScr injected mice. HSP90 used as an internal loading control. B) HMGB2 expression analysis from liver of AAV8-shHmgb2 vs AAV8-shScr injected mice. GAPDH used as an internal loading control. *36b4* is used in all qRT-PCR experiments. Data are shown as mean ± SD (n = 4) and were analyzed in (A) by Mann-Whitney test and in (B) by two-tailed unpaired t-test (*p < 0.05, **p <0.01, ***p <0.001).

Figure S8

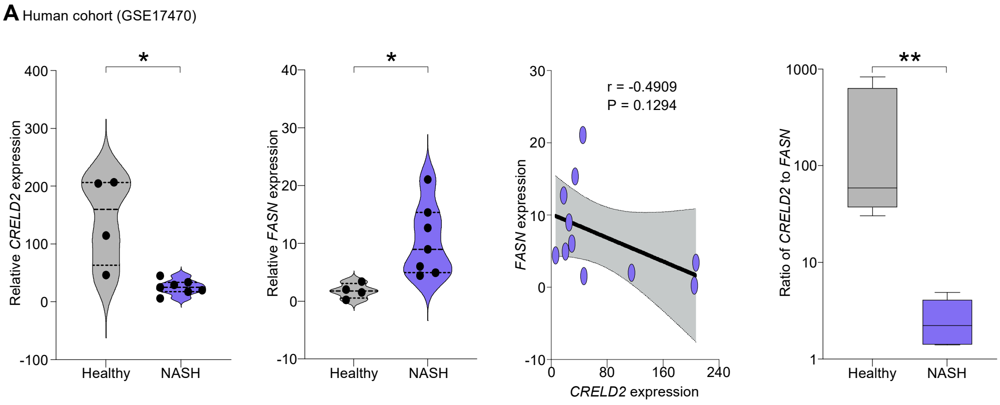

Figure S8. Negative correlation between liver *CRELD2* and *FASN* in human NASH patients. A) *CRELD2*, *FASN* gene expression along with their correlation analysis in liver of healthy (n = 4) vs NASH (n = 7) human patients. *CRELD2* to *FASN* ratio is plotted in log scale. p-value < 0.05 with the Benjamini & Hochberg method.

Figure S9

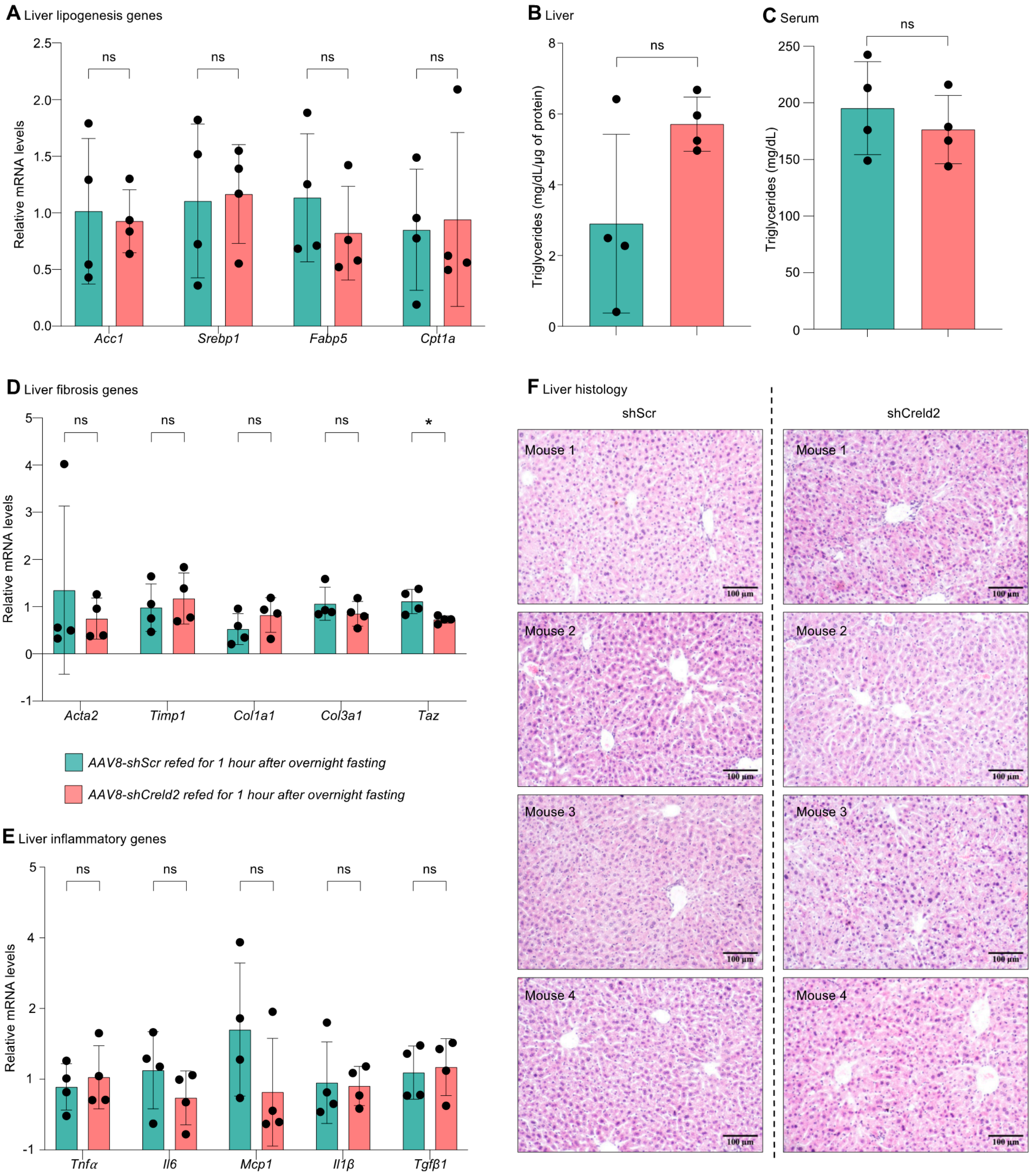

Figure S9. Comparison of AAV8 treatment in basic physiology of fast-refed mice. A) Liver lipogenesis genes expression. B, C) Liver and serum triglyceride levels. D, E) Liver fibrosis and inflammatory genes. F) Liver histology. Data are shown as mean ± SD (n = 4) and were analyzed by two-tailed unpaired t-test or Mann-Whitney test (ns: non-significant, *p <0.05). Scale bar is 100µm.

Table S1 – Composition of FPC diet.

**Food Component**	**g/kg diet**
Casein, "Vitamin-Free"	140.0
Sucrose	341.5
Maltodextrin	119.6
Vegetable shortening, hydrogenated (Primex)	190.0
Anhydrous milk fat	60.0
Palmitic acid	40.0
Cholesterol	12.5
Cellulose	50.0
Mineral mix, AIN-76 (170915)	35.0
Calcium carbonate	4.0
Vitamin mix, w/o choline, A, D, E (83171)	5.0
Vitamin E, DL-alpha tocopheryl acetate	0.1
Vitamin A palmitate	0.04
Vitamin D3, cholecalciferol	0.0044
Choline dihydrogen citrate	2.28
**Drinking water**	**g/L**
45% fructose solution (w/w)	42

Table S2 - List of mouse primers used for qRT-PCR analysis.

**Gene**	**Forward Primer**	**Reverse Primer**
Creld2	CAACACGGCCAGGAAGAATTT	CATGATCTCCAGAAGCCGGAT
36b4	GCTCCAAGCAGATGCAGCA	CCGGATGTGAGGCAGCAG
ITR	GGAACCCCTAGTGATGGAGTT	CGGCCTCAGTGAGCGA
Taz	CCCCCGCTTTGGACAGAAAAT	AGGCTGGAAATGATTGTGGAG
Acta2	GTCCCAGACATCAGGGAGTAA	TCGGATACTTCAGCGTCAGGA
Col1a1	GCTCCTCTTAGGGGCCACT	CCACGTCTCACCATTGGGG
Col3a1	CTGTAACATGGAAACTGGGGAAA	CCATAGCTGAACTGAAAACCACC

Timp1	GCAACTCGGACCTGGTCATAA	CGGCCCGTGATGAGAAACT
Fasn	GGAGGTGGTGATAGCCGGTAT	TGGGTAATCCATAGAGCCCAG
Acc1	ATGGGCGGAATGGTCTCTTTC	TGGGGACCTTGTCTTCATCAT
Srebp1	GCAGCCACCATCTAGCCTG	CAGCAGTGAGTCTGCCTTGAT
Fabp5	TGAAAGAGCTAGGAGTAGGACTG	CTCTCGGTTTTGACCGTGATG
Cpt1a	CTCCGCCTGAGCCATGAAG	CACCAGTGATGATGCCATTCT
Tnfa	CCCTCACACTCAGATCATCTTCT	GCTACGACGTGGGCTACAG
Il6	TAGTCCTTCCTACCCCAATTTCC	TTGGTCCTTAGCCACTCCTTC
Mcp1	TTAAAAACCTGGATCGGAACCAA	GCATTAGCTTCAGATTTACGGGT
Il1b	GCAACTGTTCCTGAACTCAACT	ATCTTTTGGGGTCCGTCAACT
Tgfb1	CTCCCGTGGCTTCTAGTGC	GCCTTAGTTTGGACAGGATCTG

Table S3 - List of antibodies used for western blot analysis.

Manufacturer	Cat. No.	Antibody	Dilution
R&D Systems	AF3686	Mouse CRELD2 Goat polyclonal antibody	1:1000
R&D Systems	AF954	Mouse DPPIV/CD26 Antibody	1:1000
Cell Signaling Technology	14163T	HMGB2 (D1P9V) Rabbit mAb	1:1000
Novus Biologicals	NB100-93575SS	VP1 Antibody - BSA Free Rabbit polyclonal antibody	1:1000
Sigma Aldrich	A3854	Anti-β-Actin−Peroxidase antibody	1:1000
Sigma Aldrich	G9295	Monoclonal Anti-GAPDH−Peroxidase	1:1000
Cell Signaling Technology	79641S	HSP90 (C45G5) Rabbit mAb (HRP Conjugate)	1:1000
Jackson ImmunoResearch Laboratories	705-035-003	Peroxidase AffiniPure™ Donkey Anti-Goat IgG (H+L) 2°Ab	1:5000
Jackson ImmunoResearch Laboratories	111-035-003	Peroxidase AffiniPure™ Goat Anti-Rabbit IgG (H+L) 2°Ab	1:5000

Table S4 - List of all five Sh-Creld2 clone sequences.

Clones	Forward sequence	Reverse complementary sequence
Clone 1	5’- CAC CAG ACG CTG TCT AAG TAC GAA TTT CAA GAG AAT TCG TAC TTA GAC AGC GTC -3’	5’- AAA AGA CGC TGT CTA AGT ACG AAT TCT CTT GAA ATT CGT ACT TAG ACA GCG TCT -3’
Clone 2	5’- CAC CAC CAA CCT ATT TGA GTG GTT CTT CAA GAG AGA ACC ACT CAA ATA GGT TGG -3’	5’- AAA ACC AAC CTA TTT GAG TGG TTC TCT CTT GAA GAA CCA CTC AAA TAG GTT GGT -3’
Clone 3	5’- CAC CAG ATA TAG ATG AAT GCT CAC TAT CAA GAG TAG TGA GCA TTC ATC TAT ATC -3’	5’- AAA AGA TAT AGA TGA ATG CTC ACT ACT CTT GAT AGT GAG CAT TCA TCT ATA TCT -3’
Clone 4	5’- CAC CAG AAG CCA AAC TCA CCC TTT AAT CAA GAG TTA AAG GGT GAG TTT GGC TTC -3’	5’- AAA AGA AGC CAA ACT CAC CCT TTA ACT CTT GAT TAA AGG GTG AGT TTG GCT TCT -3’
Clone 5	5’- CAC CAC CTT TAA GTT ATT GAG AGG ATT CAA GAG ATC CTC TCA ATA ACT TAA AGG -3’	5’- AAA ACC TTT AAG TTA TTG AGA GGA TCT CTT GAA TCC TCT CAA TAA CTT AAA GGT -3’

Table S5 – Composition of transformation buffer.

Manufacturer	Cat. No.	Chemical	Concentration
Sigma Aldrich	P6757	PIPES	10 mM
Sigma Aldrich	C3306	Calcium chloride dihydrate	75 mM
Sigma Aldrich	P9541	Potassium chloride	250 mM
		Dissolve in MQ water	
Sigma Aldrich	484016	Potassium hydroxide	pH changes to 6.7
Sigma Aldrich	M3634	Manganese chloride	55 mM

Table S6 – Composition of KCM buffer (5X).

Manufacturer	Cat. No.	Chemical	Concentration
Sigma Aldrich	M8266	Magnesium Chloride	250 mM
Sigma Aldrich	C3306	Calcium chloride dihydrate	150 mM
Sigma Aldrich	P9541	Potassium chloride	0.5 M

Table S7 – Composition of Terrific broth.

Manufacturer	Cat. No.	Chemical	Concentration
HiMedia	CR014	Tryptone	1.2%
Pronadisa	1702	Yeast extract	2.4%
Merck	1.07051	Glycerol	0.4%
		Dissolve in 360 mL of water and autoclave	
Merck	104873	Potassium dihydrogen phosphate	0.017 M
Merck	105109	di-Potassium hydrogen phosphate	0.072 M
		Filter sterilization	
